# Identification of Regulatory SNPs Associated with Vicine and Convicine Content of *Vicia faba* Based on Genotyping by Sequencing Data Using Deep Learning

**DOI:** 10.3390/genes11060614

**Published:** 2020-06-05

**Authors:** Felix Heinrich, Martin Wutke, Pronaya Prosun Das, Miriam Kamp, Mehmet Gültas, Wolfgang Link, Armin Otto Schmitt

**Affiliations:** 1 Breeding Informatics Group, Department of Animal Sciences, Georg-August University, Margarethe von Wrangell-Weg 7, 37075 Göttingen, Germany; felix.heinrich@uni-goettingen.de (F.H.); martin.wutke@uni-goettingen.de (M.W.); pronayaprosun.das@stud.uni-goettingen.de (P.P.D.); miriam-kamp@gmx.net (M.K.); gueltas@cs.uni-goettingen.de (M.G.); 2 Center for Integrated Breeding Research (CiBreed), Albrecht-Thaer-Weg 3, Georg-August University, 37075 Göttingen, Germany; 3 Department of Crop Sciences, Georg-August University, Von-Siebold-Str. 8, 37075 Göttingen, Germany; wlink@gwdg.de

**Keywords:** promoter, rSNP, convolutional neural network, *Vicia faba*, GBS, vicin/convicin

## Abstract

Faba bean (*Vicia faba*) is a grain legume, which is globally grown for both human consumption as well as feed for livestock. Despite its agro-ecological importance the usage of *Vicia faba* is severely hampered by its anti-nutritive seed-compounds vicine and convicine (V+C). The genes responsible for a low V+C content have not yet been identified. In this study, we aim to computationally identify regulatory SNPs (rSNPs), i.e., SNPs in promoter regions of genes that are deemed to govern the V+C content of *Vicia faba*. For this purpose we first trained a deep learning model with the gene annotations of seven related species of the Leguminosae family. Applying our model, we predicted putative promoters in a partial genome of *Vicia faba* that we assembled from genotyping-by-sequencing (GBS) data. Exploiting the synteny between *Medicago truncatula* and *Vicia faba*, we identified two rSNPs which are statistically significantly associated with V+C content. In particular, the allele substitutions regarding these rSNPs result in dramatic changes of the binding sites of the transcription factors (TFs) MYB4, MYB61, and SQUA. The knowledge about TFs and their rSNPs may enhance our understanding of the regulatory programs controlling V+C content of *Vicia faba* and could provide new hypotheses for future breeding programs.

## 1. Introduction

New methods in the field of genome sequencing—commonly summarized as next generation sequencing (NGS)—offer cost-effective strategies to produce massive amounts of sequencing data. One of these methods is genotyping-by-sequencing (GBS), which is an efficient method to obtain genome-wide genotype data for any species [1]. The characteristic feature of GBS is the reproducible generation of short genomic fragments using known restriction enzymes. Thanks to its easy applicability, GBS is currently the method of choice in the field of plant sciences since it makes plants without reference genome amenable to genomic analysis. Several groups have applied GBS to obtain high-quality genome-wide SNP markers. These markers have often been used for applications like GWAS, marker-assisted selection, breeding value estimation in genomic prediction, analysis of high density genetic maps, or assessment of population dynamics in plant genomics and plant breeding [2,3,4,5,6,7,8,9,10]. Another remarkable feature of GBS has been highlighted by Elshire et al. [11], namely that it made the analysis of regulatory regions of genes such as promoters feasible. Despite the growing interest in the analysis of GBS sequenced reads, this capacity of GBS has been poorly studied. This sequencing approach is particularly important for crop species which still often lack a reference genome sequence such as *Vicia faba*. The capacity of GBS to generate sequences of regulatory regions could be used for the prediction of such regions, e.g., promoters, in *Vicia faba*.

The faba bean is an Old World grain legume, which is grown both for combine harvested feed and as vegetable crop for human consumption. It is diploid with 2x=12 very large chromosomes. Due to its large size of 13 Gbp [12], there is thus far no sequenced and annotated reference genome available for this plant. Despite its agro-ecological importance (N-symbiosis, rotation hygiene, and pollinator support) [13] it is a crop of limited importance in many countries. This is mainly caused by its anti-nutritive seed-compounds vicine and convicine, which are co-occurring pyrimidine glycosides (in the following termed V+C) and have negative effects to animals such as laying hens, broilers and piglets, but also to 400 million humans suffering from G6PD deficiency [14,15]. The V+C content is a factor that severely limits the wider usage of *Vicia faba* as feed for animals and food for humans. Breeding V+C-poor varieties and production and marketing of their fruits could have a range of positive effects including e.g., reduction of environmentally critical soya bean imports into Europe and Northern America, fostering of regional production methods, and avoidance of energy intensive transports. To date, the location of the gene controlling the V+C content could only be restricted to a region on chromosome 1 of *Vicia faba* that exhibits conserved synteny with chromosome 2 of the related species *Medicago truncatula* between the *Medicago truncatula* genes Medtr2g008210 and Medtr2g010180 [14,16].

The promoter of a gene is the region immediately around its transcription start site (TSS) as well as further upstream of it. The promoter contains multiple elements that allow the binding of the RNA polymerase II (Pol II) along with transcription factors (TFs), thus controlling the transcription of the associated gene. Due to their impact on the gene regulation the SNPs located in the promoter regions that affect the transcription factor binding sites (TFBSs) are commonly called regulatory SNPs (rSNPs). Today it is well known that these rSNPs may be causal for the phenotype and could therefore possibly provide prime candidates useful for breeding programs or marker-assisted selection [17,18,19,20,21,22]. Despite the rich literature on the analysis of promoters, their prediction remains a challenging task due to their complex and diverse structure. Until now, different machine learning approaches have been developed, which form the core of most computational prediction methods for promoter regions. Whereas in early works the emphasis was on the identification of specific promoter elements (such as TATA boxes, initiator elements (Inrs), downstream promoter elements (DPE) and others) or extraction of k-mer distributions [23,24,25,26,27,28,29,30], nowadays a more holistic approach is given preference in that whole genomic regions are examined in Convolutional Neural Networks (CNNs), which have been successfully applied in many species [31,32,33,34,35,36].

A large scale genome-wide key study has been conducted by Kumari et al. for the prediction and analysis of core promoter elements (CPEs) across plant monocots and dicots [37]. For this purpose, CPEs of four monocots and four dicots were comprehensively analyzed and compared to establish the common as well as the specific properties of CPEs in promoter sequences. The results obtained in [37] are, on the one hand, promising to enhance the limited knowledge available about the differences between dicots and monocots with respect to their CPEs. On the other hand, they contributed to gain novel insight into the plant promoter sequence architectures and showed that some promoter signatures are strongly conserved within larger groups of plants like monocots or dicots. Based on these findings, Shahmuradov et al. developed a model, namely TSSPlant, for the prediction of plant Pol II promoters across species boundaries [38].

In line with the studies of Kumari et al. [37] and Shahmuradov et al. [38] we designed an analysis workflow for the prediction of promoter sequences as well as rSNPs of *Vicia faba* in this study. For this purpose, we first trained a CNN model using the known promoter sequences of seven plants of the Leguminosae family. Second, using GBS sequence reads of 20 *Vicia faba* lines with known V+C content, we assembled a *de novo* draft partial genome. Thereafter, we called the genomic variants by aligning the GBS reads to the partial genome to obtain high quality SNPs for candidate gene association studies. Next, applying our CNN model to the partial genome sequences, we have predicted the potential promoter sequences of *Vicia faba*. Finally, we analyzed the SNPs in these promoter sequences that were associated with the V+C content of *Vicia faba* regarding their effect on the binding affinity of TFs. Our results show that 2.46% of the assembled sequences were predicted to be promoters. We found 14 regulatory SNPs that could be mapped to the syntenic *Medicago truncatula* region harbouring the major gene for low V+C content [14,16]. These findings could be of use to increase our understanding of the regulation of the V+C content and could provide novel genomic targets for future breeding strategies of V+C poor *Vicia faba* varieties.

## 2. Materials and Methods

### 2.1. Plant Material and Sequencing

In total, 20 inbred lines of *Vicia faba* were selected of which six had low V+C content and 14 had high V+C content (see Supplementary Table S5). The lines were inbred via single-seed descent from cultivars, from a gene-bank accession, from biparental crosses or from a landrace and include winter and spring types. DNA was extracted from the grains of the plants. Two pooled grains were used per line. DNA extraction was done with LGC’s sbeadex livestock kit following the lysis protocol L for plant tissue. Genotyping-by-sequencing was carried out on the Illumina NextSeq 500 V2 platform. The DNA was digested with the restriction enzyme MslI (recognition sequence: CAYNN⌃NNRTG). Per sample ∼3 million 150 bp paired end reads were obtained. Then sequencing adapter remnants were clipped and reads whose 5${}^{\prime }$ ends did not match the restriction enzyme site were discarded. The sequencing and filtering was performed by LGC Genomics GmbH (Berlin, Germany).

### 2.2. Assembly of a Partial *Vicia faba* Genome

Following the *de novo assembly* strategies used in [39,40], we applied the *de novo* assembler Trinity [41] to the GBS sequence reads for the construction of a partial genome for *Vicia faba*. In total, 694,605 contigs with an average length of 236 bp were constructed. To filter out redundant contigs, we clustered the contigs with CD-HIT [42] using a threshold of 95.0% for sequence identity.

### 2.3. Variant Calling and Association Testing

Following the variant calling pipeline outlined in [43], we mapped the sequence reads onto the partial genome using Bowtie2 [44]. The variant calling was done with SAMtools mpileup [45]. We excluded structural variants such as insertions and deletions as well as non-biallelic SNPs yielding 1,880,592 SNPs. Low quality SNPs with a quality score of lower than 400 were excluded using PLINK 1.9 [46]. The association between candidate SNPs and the V+C content was tested with PLINK using a 1df chi-squared allelic test. To control the type I error rate we set the false discovery rate (FDR) to 0.1.

### 2.4. Data Sets for Training the Neural Network

Mainly considering the members of the Leguminosae family, we used in our analysis seven species (*Glycine max*, *Lupinus angustifolius*, *Medicago truncatula*, *Phaseolus vulgaris*, *Trifolium pratense*, *Vigna angularis*, and *Vigna radiata*) that have a complete and annotated reference genome sequence available. To further establish the cross-species promoter prediction performance of our CNN model with a more distant plant, we also chose to include in our analysis the model species *Arabidopsis thaliana*. Following [31,33], we extracted for each species their core promoter sequences covering the −200 bp to +50 bp regions relative to the transcription start sites (TSSs) of protein coding genes from the Ensembl Plants database (release 45) [47] using BioMart [48]. Simultaneously, the sequences covering [TSS+751,TSS+1000] from the core gene region of the genes were extracted, as non-promoter sequences. Sequences that were not assigned to a chromosome or which contained ambiguous bases were not considered.

Currently, due to the absence of an annotated reference genome, there is only scarce knowledge about the promoter sequence architecture in *Vicia faba*. Hence, it is still challenging to determine characteristic signatures of *Vicia faba* promoters that distinguish them from non-promoter regions. To eliminate this lack of knowledge to some extent and to enhance the distinction of promoters vs. non-promoters in *Vicia faba*, the consideration of additional non-promoter sets is important. Consequently, we included two further sets of sequences of length 250 bp as non-promoters in our analysis. While the first set was randomly extracted from the *Medicago truncatula* reference genome by excluding the region [TSS-1000,TSS+500], the second set was sampled from the *Vicia faba* reference transcriptome V2 which was downloaded from the Pulse Crop Database [49]. The final number of sequences for each data set can be found in Table 1.

### 2.5. Sequence Features Used to Predict Promoters

In line with previous studies [33,38], we used a variety of additional features to characterize promoter sequences as precisely as possible. We have determined the distribution of the following features for the sequence sets listed in Table 1:

**Feature 1:** Frequency of the dinucleotides CA and CG

**Feature 2:** Frequency of the TATA motif

**Feature 3:** CG-skew of sequences ($CG_{\mathrm{skew}}=\frac{\#C-\#G}{\#C+\#G}$ where #C and #G refer to the counts of nucleotides *C* and *G* in the sequences)

**Feature 4:** Frequency of *k*-mers using different values for *k*

**Information theory based features:** we included in our analysis two additional features, namely the Horizontal Mutual Information (HMI) and the Generalized Topological Entropy (GTE).

The HMI is calculated based on a predefined distance *d* between two positions in a sequence and provides a measure of auto-covariation between the nucleotides of interest [50].

(1) $$\mathrm{HMI}\left(d\right)=\sum_{m=\{A,C,G,T\}}\sum_{n=\{A,C,G,T\}}p_{mn}\left(d\right)\cdot \log\frac{p_{mn}\left(d\right)}{p_{m}\left(d\right)p_{n}\left(d\right)},$$

where $p_{m}\left(d\right)$, $p_{n}\left(d\right)$ and $p_{mn}\left(d\right)$ refer to the marginal and joint probabilities of the nucleotides being *d* bp apart. A high value of HMI(*d*) indicates a strong correlation between the nucleotides regarding their distance *d*.

Entropy is a measure to reflect the complexity of sequences. It has been used to characterize the randomness of DNA sequences [51]. More specific varieties of entropies such as the GTE have been successfully applied in [52,53] to explore and to compare the complexity of introns, exons, and promoter regions. Based on the findings of these studies, we included GTE as an additional feature in our analysis which could provide an important information.

Let ω be a DNA sequence of length |ω| and let $n_{\omega }$ be the unique integer such that $4^{n}+n-1\leq \left|\omega \right|<4^{n+1}+\left(n+1\right)+1$. Then the GTE is defined as

(2) $$H_{n_{\omega }}^{k}\left(\omega \right)=\frac{1}{k}\sum_{i=n_{\omega }-k+1}^{n_{\omega }}\frac{\log_{4}\left(p_{\omega }\left(i\right)\right)}{i}$$

where $p_{\omega }\left(i\right)$ refers to the number of unique sub-sequences of length *i* that appear in ω. We set $k=n_{\omega }$ to consider sub-sequences of all possible lengths.

### 2.6. Convolutional Neural Networks

Our proposed model follows a CNN architecture, which is nowadays one of the most popular neural network architectures [54]. Using convolutional layers as its core elements, a CNN is able to automatically learn local as well as global features from the data layer-wise by applying a convolution operation and by encoding specific aspects of the data [55,56,57]. Within a layer, an array of stacked weight matrices of dimension W×H×D, where *W*, *H*, and *D* correspond to the width, height, and depth of the array, respectively, is moved spatially across the input data [31,34]. At every possible position, the summed element-wise product between the weight matrices and a subset of the input is calculated and a corresponding feature map is computed.

**Figure 1:**
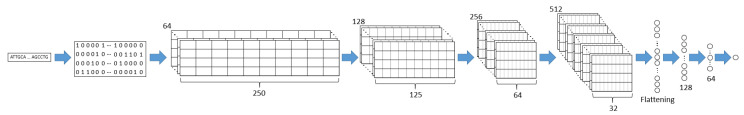
The network architecture of the CNN promoter prediction consists of four 1D-convolutional layers followed by a flattening layer and two fully-connected layers. At the end, an output layer with one neuron and a sigmoid activation function computes the probability that the analyzed sequence is classified as a promoter sequence.

The structure of the network that was used is illustrated in Figure 1. The input of the network is formed by a sequence of nucleotides of length 250 bp where each nucleotide is encoded into a one-hot representation and expressed by a four-dimensional vector, with A encoded as (1,0,0,0), C as (0,1,0,0), G as (0,0,1,0), and T as (0,0,0,1). As can be seen in Figure 1, the network is composed of four 1D-convolutional layers followed by a flattening layer, two fully-connected layers and an output layer. All convolutional layers are implemented using a ReLU activation, a stride parameter of 2, zero-padding and a filter size of 21. The first layer uses 64 filters, whereas the second, third and fourth layers use 128, 256, and 512 filters, respectively. To avoid overfitting the training data, a dropout layer with rate =0.2 is used after each convolution [58]. After processing of the sequences by the convolutional layers, a flattening layer transforms the output to a one-dimensional vector and passes its values to two consecutive fully-connected layers with 128 and 64 neurons, respectively. Finally, an output layer with a sigmoid activation classifies the input sequence as promoter or non-promoter. Additional features were included one-by-one by concatenating their values to the flattening layer in order to explore their effect on the improvement of the classification performance.

Before training the final model, a separate network for each species was trained individually using the Adam optimizer [59], L2-regularization and binary cross-entropy loss [60]. For each network, 90% of the sequences were used for model training and 10% for testing. The CNN was implemented in R using Keras [61] with TensorFlow [62] as a backend.

To assess the prediction performance we identified the number of correctly predicted promoter and non-promoter sequences as True Positives (TP) and True Negatives (TN), as well as the number of true promoter sequences predicted as non-promoter sequences, False Negatives (FN), and the number of true non-promoter sequences predicted as promoter sequences, False Positives (FP). From these measures, we calculated Accuracy (ACC), Sensitivity (true positive rate), Specificity (true negative rate), and the Matthews Correlation Coefficient (MCC) as below [33,34,63]:

(3) $$\mathrm{ACC}=\frac{TP+TN}{TP+TN+FP+FN}$$

(4) $$\mathrm{Sensitivity}=\frac{TP}{TP+FN}$$

(5) $$\mathrm{Specificity}=\frac{TN}{TN+FP}$$

(6) $$\mathrm{MCC}=\frac{TP\times TN-FP\times FN}{\sqrt{\left(TP+FP)(TP+FN)(TN+FP)(TN+FN\right)}}$$

### 2.7. Identification of Putative Regulatory SNPs

In order to identify regulatory SNPs (rSNPs), we analyzed the predicted promoter sequences of *Vicia faba*. For this purpose, we first selected all SNPs that are located in promoters and that we could successfully map against the *Medicago truncatula* genome from the initial 685,215 SNPs (see Section 2.3). Second, we extracted for each SNP its flanking sequence covering ±25 bp relative to the position of the SNP. Third, two copies of the extracted sequences were created: while the first sequence contained at the SNP position the reference allele, the second contained the alternate allele. Thereafter, we identified putative transcription factor binding sites (TFBSs) by applying the MATCH™ program [64] together with a non-redundant plant position weight matrix (PWM) library obtained from the TRANSFAC database [65] to the flanking sequences of each SNP. The MATCH™ program provides for each putative TFBS a matrix similarity score (MSS) ranging from zero to one, which reflects the potential binding affinity of the related TF to it. Finally, we predicted the consequence of each SNP on the TFBS by comparing their MSSs in the two sequences. As a result we observed in our analysis four different types of consequences: (i) no effect, (ii) change in binding affinity, (iii) loss of TFBS (a TFBS appears only for the reference allele) and (iv) gain of TFBS (a TFBS appears only for the alternate allele). Two TFBSs are considered as identical if their PWMs, positions and their strands are equal for both alleles. If the scores computed by MATCH™ are identical in both alleles, the SNP is assumed to have no effect on the TFBS. In our further analysis, we define a SNP as a rSNP, if it has an effect on the binding affinity of at least one TF, i.e., if its type of consequence is (ii), (iii), or (iv).

## 3. Results and Discussion

Classical application of GBS includes the identification and genotyping of large numbers of genomic variants. This provides several possibilities in plant breeding like the discovery of important markers by GWAS even in the absence of the reference genome. In this study, however, we focused on another important property of the GBS approach, namely its capacity to access regulatory regions (especially promoters) which serves as a basis for the identification of rSNPs in *Vicia faba*.

### 3.1. Processing the GBS Data

Sequencing of the 20 *Vicia faba* samples yielded 51 GB of GBS data. The *de novo assembly* and filtering resulted in a partial genome consisting of 419,390 contigs with a total length of 100,037,292 bp. Considering that the proposed size of the *Vicia faba* genome is about 13 Gbp [12] our partial genome covered 0.77% of the total genome. Through remapping of the reads to the partial genome with Bowtie2 and subsequent variant calling with SAMtools 1,880,592 SNPs could be derived. The quality scores of these SNPs as given in the vcf file showed a clear bimodal distribution with a minimum at a quality score of 400. 1,195,377 SNPs having a quality score of lower than 400 were discarded, such that 685,215 high quality SNPs remained.

### 3.2. Prediction of Promoter Sequences

#### 3.2.1. Intra- and Inter-Species Promoter Prediction

In order to gain first insights into the predictability of promoters of the seven Leguminosae family members and *Arabidopsis thaliana*, we trained our CNN model for each species individually. The prediction reliability of the CNN model has been examined for each species by classifying the intra- and inter-species promoters that were not used in the training process. To assess the performance of the classification, the ACC, Sensitivity, Specificity and MCC values were calculated. The details based on the ACC values are presented in Table 2 and the results based on the remaining measures are given in the Supplementary Tables S1–S3.

The results presented in Table 2 show that although the CNN models have been trained only using one-hot representation of sequences for each species individually, the network architecture is able to recognize certain patterns in the sequences which leads to the predictability of promoters across different species to a certain degree. These findings support the results presented in [37] and indicate that some of the promoter signatures seem to be conserved between the *Leguminosae* family members.

Further, Table 2 demonstrates that the classification performance of some CNN models remarkably results in higher ACC values for inter-species prediction than for intra-species prediction. In particular, this is the case for the species *Lupinus angustifolius* and *Vigna angularis* whose promoters have been predicted with very high accuracy by the CNN models of the other species except for the *Arabidopsis thaliana* and *Trifolium pratense* models. This could be attributed to the underlying genome annotations of these species since their annotations seem to be created based on the genome information of well-studied family members. Especially, this assumption is true regarding the inclusion of the Leguminosae family specific promoter patterns in the promoters of these species. This hypothesis has been supported by the prediction performance of the *Lupinus angustifolius* model on the other species, which reached very high degrees of specificity while only achieving low degrees of sensitivity. To this end, we compared the inter-species prediction ACC values of the species regarding their performance on *Lupinus angustifolius* and *Vigna angularis*. This comparison revealed that the promoters of *Lupinus angustifolius* were predicted with a slightly higher mean accuracy value than the promoters of *Vigna angularis* (0.880 compared to 0.868).

So far, we trained our CNN model only using the order of the nucleotides in DNA sequences (one-hot encoding). However, previous studies pointed out that the combination of one-hot encoding with additional widely used features could lead to a substantially improved performance in promoter identification [33,34]. For this purpose, we followed a similar procedure as suggested by Triska et al. in [33] and systematically evaluated the combination of sequence features (see Section 2.5) in the CNN model training of each species. The results are presented only for *Medicago truncatula* as an example in Table 3.

In contrast to previous studies [33,34], Table 3 shows that regardless of the usage of any additional feature, the performance of the CNN model could in general not be significantly improved. However, these results are in agreement with findings presented in [31,32,35,36] and indicate that the CNN architecture is able to learn specific patterns inherent in the sequences automatically. Hence, these patterns carry information which is obviously redundant to these widely used features. Consequently, it turns out that the consideration of additional features does not lead to an improvement of the CNN model performance and may, on the contrary, increase the noise during training.

#### 3.2.2. Prediction of *Vicia faba* Promoters

The knowledge about the promoter signatures which are conserved between the *Leguminosae* family members provides an important clue for the precise prediction of *Vicia faba* promoters, which still remains a challenge. However, the consideration of the sequences of only one *Leguminosae* family member in the CNN model could be insufficient to capture the variety of different promoter signatures for the accurate computational identification of the *Vicia faba* promoters. To mitigate the drawback of single species models we systematically examined different CNN models seeking to determine the preferential combination of *Leguminosae* family members by intensifying the signal of promoter sequences and thus to improve the performance of the CNN model. Consequently, we trained a CNN model based on the species *Lupinus angustifolius* and *Medicago truncatula* since the combined usage of their manually selected sequences perfectly complement each other. In the last step, we included in the CNN model training two additional non-promoter sets (defined in Section 2.4) to enhance the distinction signals between promoter and non-promoter regions. The training sequences are given in the Supplementary Files S6 and S7. The evaluation of this CNN model yields to clearly better ACC and MCC values of 0.98 and 0.95, respectively. A further analysis reveals that the usage of other sequence features together with one-hot encoding in our final CNN model does not affect the performance of the classifier.

Finally, by applying the CNN model to the *Vicia faba* sequences of length 250 bp, we classified in total 2.46% of them as potential promoter sequences. It is important to note that, due to the random fragment orientation regarding the direction of the reads from GBS, the correct direction of the sequences in the *de novo* assembly draft partial genome of *Vicia faba* is unknown. To address this limitation, we considered in our predictions four different types of the sequences as: (i) the original obtained assembly; (ii) the complement of the obtained assembly that is gained by keeping the reading direction; (iii) the reverse of the obtained assembly that is gained by changing the reading direction; and (iv) the reverse complement of the obtained assembly.

Checking the positions of the SNPs in the contigs disclosed that in total 132,399 out of 685,215 SNPs were located in the predicted *Vicia faba* promoters. A flanking sequence of ±25 bp could only be obtained for 118,492 SNPs. These SNPs with a complete flanking sequence were mapped against the *Medicago truncatula* genome using the BLASTN algorithm with a threshold of 0.01 for the *e*-*value* and of 0.9 for the *percent identity* [66]. Overall, we found 33,846 hits for 1976 SNPs showcasing the repetitiveness of the *Medicago truncatula* genome. We identified 14 SNPs that map to the predefined target region of *Medicago truncatula* that harbours orthologous genes associated with the V+C content of *Vicia faba* [14,16]. This target region is ranging approximately from 1,300,000 bp to 2,300,000 bp of the *Medicago truncatula* chromosome 2. An overview of these SNPs and their mapped position in the *Medicago truncatula* genome along with the genes with the closest TSS is given in Table 4. We tested these 14 SNPs for their association with the V+C content with PLINK. The adjusted *p*-values presented in Table 4 suggest that SNP_341016_236 and SNP_341016_239, which are located in the same promoter, show a highly significant association with the V+C content in *Vicia faba* while the associations of the remaining SNPs are not significant at the level α=0.05. For both of these SNPs the reference allele only occurs in the low V+C lines with one exception while the alternate allele is restricted to the high V+C lines (see Supplementary Table S8).

#### 3.2.3. Systematic Identification of Regulatory SNPs Associated with the V+C Content of *Vicia faba*

Following the studies of Xu et al. and Fu et al. in [67,68], we scanned the flanking sequences of the SNPs by applying the MATCH™ program [64] to systematically identify the SNPs that are likely to affect the binding affinity of transcription factors (TFs) and, thus, influence the gene expression level. This search was done for the 1976 SNPs that were located in the predicted *Vicia faba* promoters and which could be successfully mapped onto the *Medicago truncatula* genome. We considered results of this run with an MSS score ≥0.85 as putative TFBSs (as suggested in [69]). 9444 putative TFBSs were identified. SNPs that were located in putative TFBSs were considered as rSNPs. Their consequence types were determined by examining their predicted effects on the binding affinities of the TFs. The rSNPs, their consequence and related TFs with corresponding PWM names are given in Supplementary Table S4. The analysis of the 14 SNPs presented in Table 4 reveals that the binding affinities of 44 TFs to their 79 TFBSs were affected. Focusing on the two highly significant SNPs (SNP_341016_236 and SNP_341016_239) in the same promoter, we found that a nucleotide substitution in SNP_341016_236 is likely to entail severe consequences regarding TF binding affinity, namely loss and gain of TFBSs.

The substitution in SNP_341016_239 results in only a moderate change of the binding affinities of TFs (see Table 5). The remarkably different consequences of both SNPs indicate their considerably different influence for the precise and effective regulation of the corresponding gene, although their *p*-values are the same.

### 3.3. Functional Analysis of the Candidate Gene and Transcription Factors

The *Medicago truncatula* gene MTR_2g008620 is the gene which is located closest to the two highly significant SNPs. It is a beta-hydroxyacyl-ACP-dehydratase that is involved in the elongation of fatty acids as well as in the related metabolism of biotin [70,71]. A direct association with the creation of V+C which has been linked to the orotic acid pathway [72] is not obvious. This seems plausible since *Medicago truncatula* does not synthesize V+C. Of more interest are the transcription factors for which we found putative binding sites that are affected by the two SNPs. The TF SQUA belongs to the MADS-box domain group whose genes play vital roles in multiple aspects of plant development (for instance development of flowers, fruits and roots as well as regulating flowering time) [73,74]. Such genes regulate, for example, stem growth and early flowering in soybean [75] or vernalization response in wheat [76]. SQUA itself is involved in the determination of floral meristem and organ identity [77,78]. The MYB domain group is one of the largest families of TFs in plants. Its members are involved in the regulation of development, metabolism, the circadian rhythm, and responses to biotic and abiotic stresses in plants [74,79,80]. In *Medicago truncatula* multiple MYB TFs including MYB4 and MYB61 are involved in flavonoid biosynthesis during macrosclereid cell development [81]. MYB4 in particular regulates abiotic stress responses towards UV-B light and cadmium toxicity in *Arabidopsis thaliana* [82,83] and cold in *Oryza sativa* [84]. It has also been shown to influence the biosynthesis of flavonoids [85]. MYB61 participates in the response to cold stress in *Medicago truncatula* [86]. In *Arabidopsis thaliana*, this TF is expressed in sink tissues, such as xylem, roots and developing seeds, and controls resource allocation influencing growth and development of the plant [87]. It has also been shown to affect trichome initiation, root development and stomatal aperture and it is necessary for the biosynthesis of gibberellin [88,89]. Furthermore, it is required for the seed coat mucilage deposition during the development of the seed coat epidermis [90,91]. This is a promising result considering that the seed coat is the suggested site of biosynthesis of the V+C compounds [92].

## 4. Conclusions

With their anti-nutritive effects the high vicine and convicine content has so far restricted the usage of *Vicia faba* as feed for livestock or as crop for human consumption. Identifying causal markers and understanding the mechanisms of regulation of the V+C content are important steps for breeding new cultivars with lower V+C content. This task is even more challenging since a complete and annotated reference genome for *Vicia faba* is still missing. In this work we harnessed the knowledge about regulatory regions in related species to train a convolutional neural network, which allowed us to predict those regions in *Vicia faba*. This model permitted us to classify DNA sequences as promoter or non-promoter without undertaking the considerable effort of assembling and annotating a reference genome. We applied this model to GBS data of *Vicia faba* for the identification of its putative promoter regions as well as regulatory SNPs therein. Our results show that we were able to detect two rSNPs significantly associated with the V+C production in *Vicia faba*. We suggest these rSNPs as promising candidates for marker-assisted selection. In particular, the associated transcription factor MYB61 could provide new insights into the molecular mechanisms underlying V+C. To the best of our knowledge, this is the first study which uses the gene annotations of related species of the *Leguminosae* family to predict promoters and rSNPs of *Vicia faba* based on the GBS data. The analysis approach that we presented here could potentially also be applied to other species that lack a reference genome which is still the case for many crop species.

## Figures and Tables

**Table 1:** Number of promoter and non-promoter sequences in the sets that were used as training sets.

Species	# Promoter Sequences	# Non-Promoter Sequences
*Arabidopsis thaliana*	23,315	23,315
*Glycine max*	46,199	46,199
*Lupinus angustifolius*	23,463	23,463
*Medicago truncatula*	32,158	32,158
*Phaseolus vulgaris*	22,750	22,750
*Trifolium pratense*	14,749	14,749
*Vigna angularis*	19,584	19,584
*Vigna radiata*	15,495	15,495
*Medicago truncatula* (Genome-wide)	-	∼12,000
*Vicia faba* (Transcriptome)	-	∼60,000

**Table 2:** ACC values of the intra- and inter-species promoter classification using the species-specific trained CNNs. Off-diagonal numbers are ACC values for inter-species classification, diagonal numbers are ACC values for intra-species classification. For instance, a CNN trained on *Lupinus angustifolius* and used for classification of *Vigna angularis* promoters has an accuracy of 0.974.

	Evaluated	*Arabidopsis* *thaliana*	*Glycine* *max*	*Lupinus* *angustifolius*	*Medicago* *truncatula*	*Phaseolus* *vulgaris*	*Trifolium* *pratense*	*Vigna* *angularis*	*Vigna* *radiata*
Trained									
*Arabidopsis* *thaliana*		0.901	0.767	0.690	0.746	0.797	0.765	0.633	0.733
*Glycine* *max*		0.837	0.864	0.915	0.847	0.863	0.724	0.914	0.856
*Lupinus* *angustifolius*		0.545	0.611	0.981	0.720	0.586	0.493	0.974	0.709
*Medicago* *truncatula*		0.755	0.797	0.959	0.876	0.789	0.715	0.951	0.841
*Phaseolus* *vulgaris*		0.845	0.842	0.888	0.834	0.898	0.748	0.880	0.853
*Trifolium* *pratense*		0.822	0.764	0.696	0.751	0.794	0.840	0.689	0.736
*Vigna* *angularis*		0.510	0.607	0.971	0.715	0.583	0.494	0.977	0.712
*Vigna* *radiata*		0.741	0.812	0.937	0.827	0.825	0.675	0.928	0.904

**Table 3:** Contribution of additional features in the CNN model of *Medicago truncatula*.

Features	Accuracy	Sensitivity	Specificity	MCC
DNA sequence	0.876	0.897	0.855	0.750
DNA sequence + 2-mer	0.874	0.880	0.867	0.747
DNA sequence + 2-mer + frequency of CA motif	0.862	0.828	0.897	0.726
DNA sequence + 2-mer + frequency of CG motif	0.875	0.875	0.876	0.751
DNA sequence + 2-mer + HMI	0.874	0.882	0.865	0.747
DNA sequence + 2-mer + frequency of TATAA motif	0.876	0.875	0.878	0.752
DNA sequence + 2-mer + CG skew	0.876	0.889	0.863	0.753
DNA sequence + topological entropy	0.874	0.886	0.861	0.747
DNA sequence + 2-mer + topological entropy	0.871	0.852	0.890	0.743
DNA sequence + 2-mer + HMI + frequency of TATAA motif	0.871	0.869	0.874	0.743
DNA sequence + 2-mer + HMI + frequency of CA motif + frequency of CG motif + frequency of TATAA motif + CC skew	0.873	0.859	0.888	0.747
DNA sequence + HMI + frequency of CA motif + frequency of CG motif + frequency of TATAA motif + CG skew	0.875	0.889	0.860	0.749

**Table 4:** The 14 SNPs found in the predicted promoters of *Vicia faba* that were mapped to the *Medicago truncatula* target genomic region.

SNP_ID	Genotype	FDR	Position	Medicago Gene
SNP_131938_118	C/T	0.234	1,385,390	MTR_2g008290
SNP_302904_183	G/A	0.179	1,385,444	
SNP_341016_236	C/T	$1.17\cdot 10^{-7}$	1,554,857	MTR_2g008620
SNP_341016_239	G/A	$1.17\cdot 10^{-7}$	1,554,860	
SNP_356745_200	A/G	0.730	1,707,078	MTR_2g008960
SNP_280549_41	C/T	0.234	1,707,183	
SNP_350273_103	G/T	0.234	1,707,199	
SNP_350273_90	A/C	0.234	1,707,212	
SNP_350273_61	G/A	0.234	1,912,704	MTR_2g009430
SNP_29452_204	G/A	0.730	1,912,812	
SNP_29452_206	G/A	0.496	1,912,814	
SNP_118828_190	C/T	0.234	2,030,017	MTR_2g009690
SNP_80231_27	C/T	0.234	2,163,048	MTR_2g009940
SNP_364434_97	A/T	0.359	2,163,084	

Genotype refers to the reference and alternative alleles; FDR is the false discovery rate obtained in an association test with the V+C content of 20 *Vicia faba* lines; Position is the position in bp on the *Medicago truncatula* chromosome 2.

**Table 5:** The two SNPs with the strongest association to the V+C content and their consequences. The column **Allele** indicates for which allele of the SNP the binding site was found. **TFBS** refers to the name of the binding sites, which were named after their PWMs. The structure of the PWM names is given as: P$TFname_version, where “P$” stands for the PWMs used for the prediction of the TFBSs of plant TFs. “TFname” refers to the name of the transcription factor, and “_version” refers to the version of the PWM.

SNP_ID	Allele	TFBS	MSS	Consequence
SNP_341016_236	Ref	P$MYB4_01	0.945	Loss of TFBS
SNP_341016_236	Ref	P$MYB61_01	0.880	Loss of TFBS
SNP_341016_236	Alt	P$SQUA_01	0.870	Gain of TFBS
SNP_341016_239	Ref	P$MYB61_01	0.880	Score change
SNP_341016_239	Alt	P$MYB61_01	0.881	Score change

## Supplementary Materials

The following are available online at https://www.mdpi.com/2073-4425/11/6/614/s1, Table S1: Sensitivity values of the interspecies promoter classification using the intraspecies trained CNNs. Table S2: Specificity values of the interspecies promoter classification using the intraspecies trained CNNs. Table S3: MCC values of the interspecies promoter classification using the intraspecies trained CNNs. Table S4: Results of the TFBS analysis. Table S5: Overview of the 20 *Vicia faba* lines used in the analysis. File S6: Promoter sequences used for training the model. File S7: Non-promoter sequences used for training the model. Table S8: Alleles of the 20 *Vicia faba* lines for the two significantly associated rSNPs.

## Abbreviations

The following abbreviations are used in this manuscript:

SNP	Single Nucleotide Polymorphism
rSNP	Regulatory Single Nucleotide Polymorphism
CNN	Convolutional Neural Network
TF	Transcription Factor
TFBS	Transcription Factor Binding Site
V+C	Vicin and Convicine
FDR	False Discovery Rate
HMI	Horizontal Mutual Information
GTE	Generalized Topological Entropy
CPE	Core Promoter Element
PWM	Position Weight Matrix
MSS	Matrix Similarity Score
